# The Hypertensive Effect of Amphotericin B-Containing Liposomes (Abelcet) in Mice: Dissecting the Roles of C3a and C5a Anaphylatoxins, Macrophages and Thromboxane

**DOI:** 10.3390/biomedicines10071764

**Published:** 2022-07-21

**Authors:** Erik Őrfi, László Hricisák, László Dézsi, Péter Hamar, Zoltán Benyó, János Szebeni, Gábor Szénási

**Affiliations:** 1 Institute of Translational Medicine, Semmelweis University, 1094 Budapest, Hungary; rickrster@gmail.com (E.Ő.); hricisak.laszlo@med.semmelweis-univ.hu (L.H.); dr.dezsi.laszlo@gmail.com (L.D.); hamar.peter@med.semmelweis-univ.hu (P.H.); benyo.zoltan@med.semmelweis-univ.hu (Z.B.); jszebeni2@gmail.com (J.S.); 2 SeroScience Ltd., 1089 Budapest, Hungary; 3 Institute for Translational Medicine, Medical School, University of Pécs, 7624 Pecs, Hungary; 4 Department of Nanobiotechnology and Regenerative Medicine, Faculty of Health Sciences, Miskolc University, 3515 Miskolc, Hungary

**Keywords:** infusion reaction, cobra venom factor, platelets, granulocytes

## Abstract

Liposomal amphotericin B (Abelcet) can cause infusion (anaphylactoid) reactions in patients whose mechanism is poorly understood. Here, we used mice to investigate the role of complement (C) receptors and the cellular sources of vasoactive mediators in these reactions. Anesthetized male NMRI and thromboxane prostanoid receptor (TP) or cyclooxygenase-1 (COX-1)-deficient and wild type C57Bl6/N mice were intravenously injected with Abelcet at 30 mg/kg. Mean arterial blood pressure (MABP) and heart rate (HR) were measured. In untreated mice, Abelcet caused a short (15 min) but large (30%) increase in MABP. C depletion with cobra venom factor (CVF) and inhibition of C5a receptors with DF2593A considerably prolonged, while C3aR inhibition with SB290157 significantly decreased the hypertensive effect. Likewise, the hypertensive response was abolished in COX-1- and TP-deficient mice. CVF caused a late hypertension in TP-deficient mice. Both macrophage depletion with liposomal clodronate and blockade of platelet GPIIb/IIIa receptors with eptifibatide prolonged the hypertensive effect. The early phase of the hypertensive effect is COX-1- and TP-receptor-dependent, partly mediated by C3aR. In contrast, the late phase is under the control of vasoactive mediators released from platelets and macrophages subsequent to complement activation and C5a binding to its receptor.

## 1. Introduction

Hypersensitivity reactions (HSRs) limit the use of intravenously administered nanomedicines, biologicals, and diagnostic agents in a small percentage of sensitive patients. Since the symptoms of HSRs are present already at the first administration without a role of IgE, it is considered as a pseudoallergy or, in serious cases, an anaphylactoid reaction. The leading symptoms of pseudoallergy are mild-to-medium circulatory changes, chest and back pain, dyspnea, coughing, fever, flushing, rash, urticaria, and many other common symptoms that characterize acute allergy [1].

There are two explanations for the mechanism of pseudoallergy or anaphylactoid reaction. Complement (C) activation has been shown to be partly responsible for HSRs leading to the term complement activation-related pseudoallergy (CARPA) [1]. The CARPA mechanism has repeatedly been confirmed in various species. However, in few studies no changes in plasma C component concentrations could be detected despite the development of the usual pseudoallergy symptoms raising the possibility of C-independent pseudoallergy (CIPA) [2]. The term CARPA was coined to emphasize that in most cases the innate immune system contributes to the side effects caused by intravenous treatments that appear at the first administration and otherwise are called hypersensitivity reactions or infusion reactions, which in few cases can lead to anaphylaxis.

There are several animal models and in vitro assays used to study the mechanisms of pseudoallergy or anaphylactoid reactions. Pigs are as sensitive as sensitive humans, making them suitable to predict nanodrug-induced symptoms in humans [3]. Rats and mice are less sensitive than pigs, but they can be used to study the molecular and cellular mechanisms due the easier experimentation and availability of transgenic animal strains. In a recent study we have shown that the liposomal formulations of amphotericin B, AmBisome, and Abelcet, as well as the direct C activators, cobra venom factor (CVF), and zymosan uniformly increased MABP for up to 20 min in anesthetized mice [2]. These changes were transient and were turned around by CVF and zymosan to lead into delayed hypotensive shock. These results seem to suggest that the hypertensive response can be considered a pseudoallergy, while, in the case of stronger stimuli caused by zymosan and CVF leading to hypotensive shock, is an anaphylactoid response. This distribution of allergy in the mouse is similar to the general practice in humans, as anaphylaxis is considered to be the most severe acute allergic disease [4]. The hypertensive effect was due to C activation with C3a release and binding to C3aR, as the blood level of the anaphylatoxin paralleled the rise of MABP [2]. Concerning the mechanism of blood pressure (MABP) changes it has been repeatedly shown that stimulation of anaphylatoxin receptors C3aR and C5aR can stimulate liberation of vasoactive mediators that alter MABP [5,6]. Activation of C5aR causes hypotension, while stimulation of C3aR can induce hypertension in several rodent species [5,6].

Liposome-encapsulated hemoglobin increased plasma thromboxane B2 (TXB2) concentration in mice [7], pigs [8], and rats [9]. TXB2 is a stable metabolite of TXA2, a strong vasoconstrictor [10]. Indomethacin, a COX-1 inhibitor abolished the hypertensive effect of recombinant human C3a in anesthetized rats [6], and attenuated the zymosan-induced increase in right ventricular systolic pressure and exaggerated hypotension, which are characteristic symptoms of CARPA in rats [11].

The current study focused on the effects of Abelcet, a liposomal formulation of amphotericin B, which was most effective in triggering a blood pressure effect in mice [2] and rats [12], and are known to cause HSRs in man in a relatively high percentage (>10%) [13,14,15]. The aim of our study was to reveal the mechanisms of Abelcet-induced changes in MABP by depletion of C with CVF and by inhibiting C3aR and C5aR receptors, and by evaluating the contribution of platelets, mast cells, and macrophages to the hypertensive effect of Abelcet. Our experiments also addressed the question whether the MABP effect of Abelcet is partly or fully mediated by the release of cyclooxygenase 1 (COX-1) products by measuring blood pressure in COX-1 and the thromboxane prostanoid (TP) receptor deficient mice.

## 2. Materials and Methods

### 2.1. Chemicals, Liposomes, and ELISA Kits

Abelcet, an amphotericin B containing liposomal preparation, and eptifibatide (Iloprost), a platelet glycoprotein IIb/IIIa receptor inhibitor, were obtained from Semmelweis University Pharmacy (Budapest, Hungary). Cobra venom factor (CVF) was obtained from TECOMedical (Sissach, Switzerland). SB290157, a C3a receptor (C3aR) antagonist, and DF2593A, a C5a receptor (C5aR) antagonist, were purchased from Merck Hungary Zrt. (Budapest, Hungary). Clodronate liposomes, a macrophage depleting preparation [16], was supplied by Liposoma BV (Amsterdam, The Netherlands).

Abelcet liposomal formulation contains l-α-dimyristoylphosphatidylcholine (DMPC), l-α-dimyristoylphosphatidylglycerol (sodium and ammonium salts) (DMPG), and saline. Amphotericin B forms a complexed ribbon-like structure with the phospholipids, which results in an approximately 100-fold decrease in amphotericin B toxicity in the rat [12].

### 2.2. Animals

A part of the study was performed on an outbred mouse strain originally developed at the Naval Medical Research Institute (Crl: NMRI BR). SPF male NMRI mice weighing 25–29 g were purchased from Toxicoop Ltd. (Budapest, Hungary). The experiments were started after a minimum of one-week adaptation following arrival.

Cyclooxygenase-1 (COX-1)-deficient mice were provided by Professor Ingvar Bjarnason (King’s College Hospital, London, UK) and were backcrossed to C57Bl6/N mice more than 10 times. Thromboxane prostanoid receptor (TP)-deficient mice were obtained from Dr. Shuh Narumiya (Kyoto University, Kyoto, Japan) and were backcrossed to C57BL/6 more than 10 times. The COX-1- and TP-deficient phenotype was confirmed before the mice were used in the experiments. Mice had free access to standard rodent chow (Altromin standard diet, Germany) and tap water.

### 2.3. Experimental Protocol

Mice were anesthetized with pentobarbital (90 mg/kg i.p.), and additional small doses were administered if needed. The right carotid artery and the left jugular vein were cannulated with PP10 tubing for measuring MABP and for drug administration, respectively. MABP was measured using a BPR-02 pressure transducer (Experimetria Ltd., Budapest, Hungary), an HG-01D BP amplifier (Experimetria Ltd., Budapest, Hungary), and a PowerLab data acquisition system (ADInstruments Ltd., Oxford, United Kingdom). MABP and heart rate (HR) were derived from the pulsatile blood pressure curve, monitored and recorded on a desktop computer using LabChart data analysis software (version 8, ADInstruments Ltd., Oxford, United Kingdom). MABP and HR were continuously recorded during the experiment, which started 10 min prior to i.v. injection of test materials (within 1 min) in a volume of 10 mL/kg. Then, 5 min after administration of drugs Abelcet was infused i.v. over 1 min via the jugular vein catheter at the dose of 30 mg/kg in a volume of 10 mL/kg.

The following groups were studied:

Control (n = 12) treated with saline or vehicle of the drugs administered. 

Abelcet administered twice at 30 mg/kg in 10 mL/kg 30 min apart. 

Eptifibatide (n = 7) was injected at the dose of 3 mg/kg in a volume of 10 mL/kg.

CVF (n = 6), diluted in saline, was injected into the tail vein in light isoflurane anesthesia 18 and 2 h before anesthesia at doses of 30 and 100 U/kg, respectively, in a volume of 10 mL/kg. In two additional mice the plasma hemolytic complement activity was tested using the sheep red blood cell assay [2] and was found to be unmeasurably low.

SB290157 (n = 6) was administered at the dose of 10 mg/kg dissolved in DMSO (10%) and saline.

DF2593A (n = 6) was administered at the dose of 10 mg/kg dissolved in DMSO (10%) and saline

Clodronate (n = 5) or empty liposomes (N = 5) were administered intravenously in light isoflurane anesthesia at 200 mL/mouse (containing 1 mg clodronate) two days before the experiment. According to immunohistochemistry, this dose fully depleted F4/80 positive cells from the liver at the time of the experiment [Kerkovits et al., personal communication, [17]].

### 2.4. Statistical Analysis

All data presented are mean ± SEM. All results are presented as % differences in comparison of the mean of the 10 min control period. If either Brown–Forsythe test or Bartlett’s test indicated significant inhomogeneity of the data among the groups, the data were log transformed for statistical analysis. The effects of treatments were evaluated using two-way ANOVA for repeated measurements followed by Dunnett’s multiple comparisons test using GraphPad Prism version 8 for Windows (GraphPad Software, La Jolla, CA, USA).

## 3. Results

### 3.1. Effects of Two Subsequent Treatments with Abelcet

**Figure 1:**
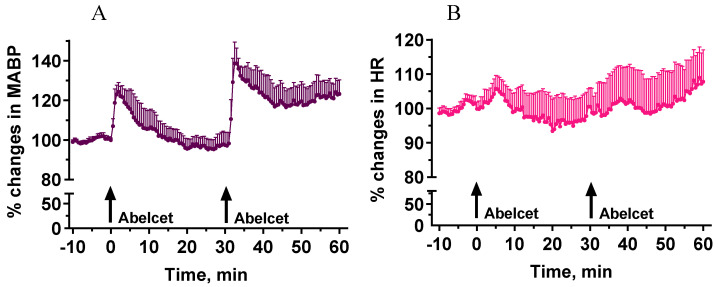
Effects of two sequential treatments with Abelcet (30 mg/kg, i.v.) on MABP and HR in anesthetized NMRI mice (n = 5). The time of i.v. Abelcet injection is indicated by arrows. (**A**): mean arterial blood pressure (MABP); (**B**): heart rate (HR). The two MABP and HR curves (for 30 min from Abelcet administration) were compared using two-way ANOVA for repeated measurements followed by Dunnett’s multiple comparison tests. MABP and HR changes were similar after the Abelcet administrations.

Administration of Abelcet at 30 mg/kg i.v. induced a significant increase in MABP (30% at its maximum) that lasted for about 15 min (Figure 1). A second treatment with Abelcet caused a similar response, although it was larger and longer to some extent, i.e., there was no tachyphylaxis to the effect of Abelcet (Figure 1). The phenomenon may be called bradyphylaxis.

### 3.2. Effects of C Depletion and C3a and C5a Receptor Antagonists on Abelcet-Induced Hypertension

**Figure 2:**
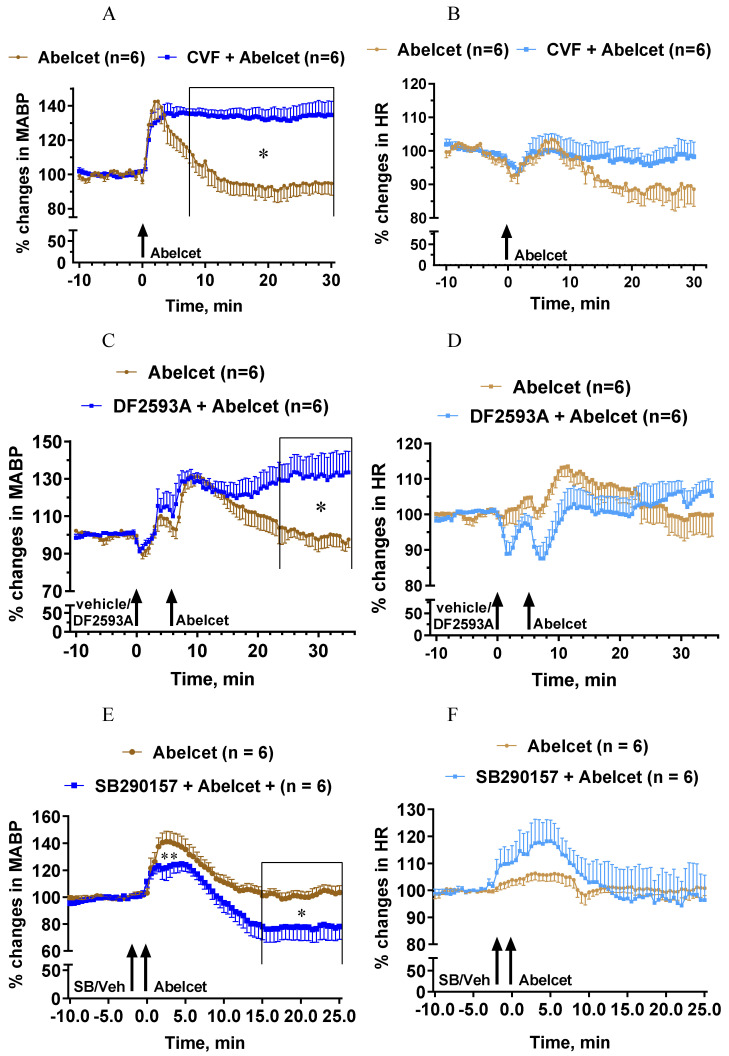
Effects of complement depletion with cobra venom factor (CVF, n = 6) and treatment with DF2593A (n = 6; C3a receptor antagonist) and SB290157 (C5a receptor antagonists; n = 6) on Abelcet-induced MABP and HR changes in anesthetized NMRI mice. The time of treatment with DF2593A, SB290157, or their vehicle and Abelcet injection is indicated by arrows. CVF was administered 18 and 2 h before anesthesia at doses of 30 and 100 U/kg, respectively, in a volume of 10 mL/kg. (**A**,**C**,**E**) Mean arterial blood pressure (MABP); (**B**,**D**,**F**) heart rate (HR). (**A**,**B**) Complement depletion with CVF; (**C**,**D**) treatment with DF2593A; (**E**,**F**) treatment with SB290157. SB: SB290157; Veh: vehicle. * *p* < 0.05. Vehicle- and CVF- or drug-treated groups were compared using two-way ANOVA for repeated measurements followed by Dunnett’s multiple comparison tests. Complement depletion with CVF lengthened the increase in MABP from min 8 after Abelcet administration, but did not alter HR. Treatment with DF2593A lengthened the increase in MABP from min 24 after Abelcet administration but did not alter HR. Treatment with SB290157 attenuated the increase in MABP at 1 and 2 min and from 15 min after Abelcet administration but did not alter HR.

Complement depletion with CVF also had a bradyphylactic effect inasmuch as it considerably lengthened the Abelcet-induced hypertension (Figure 2A). The MABP remained steadily elevated up to 30 min after treatment with Abelcet. On the other hand, complement depletion did not affect the HR response (Figure 2B). The complement C5aR antagonist, DF2593A, had an effect similar to complement depletion, as from 18 min after Abelcet administration the MABP was still significantly higher compared to Abelcet alone (Figure 2C). Again, this C5aR antagonist had no influence on the HR response to Abelcet (Figure 2D). In sharp contrast, the complement C3aR antagonist, SB290157, decreased the Abelcet-induced hypertension shortly after its administration, as well as 15 min later (Figure 2E), while the HR was similar in the two groups (Figure 2F). These data suggest that the two anaphylatoxins have opposite effects on MABP; while C3a is hypertonic, C5a is hypotonic in the mouse.

### 3.3. Effects of C3a Peptide Fragment (63–77) on Blood Pressure and Heart Rate

**Figure 3:**
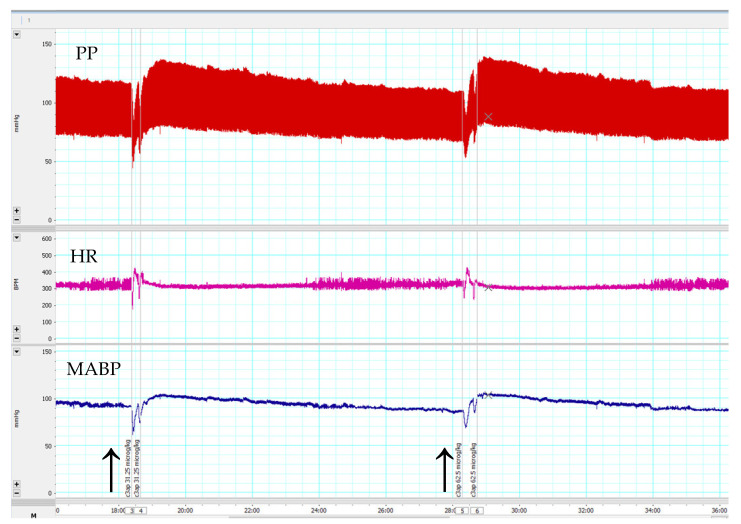
An original recording of effects of C3a (63–77) peptide at 31.25 and 62.5 µg/kg bolus i.v. doses on blood pressure and heart rate in anesthetized C57Bl/6n mice. Arrows indicate the time of C3a (63–77) peptide administrations. **Upper chart**: pulsatile blood pressure (PP); **Middle chart**: heart rate (HR); **Lower chart**: mean arterial blood pressure (MABP). C3a (63–77) peptide caused a transient increase in MABP and HR.

Consistent with the above results with the receptor antagonists, the C3a mimetic peptide fragment (63–77) caused a dose-dependent, but short-lived increase in MABP, but no changes in HR (Figure 3).

### 3.4. Effects of Macrophage Depletion and Platelet Inhibition 

**Figure 4:**
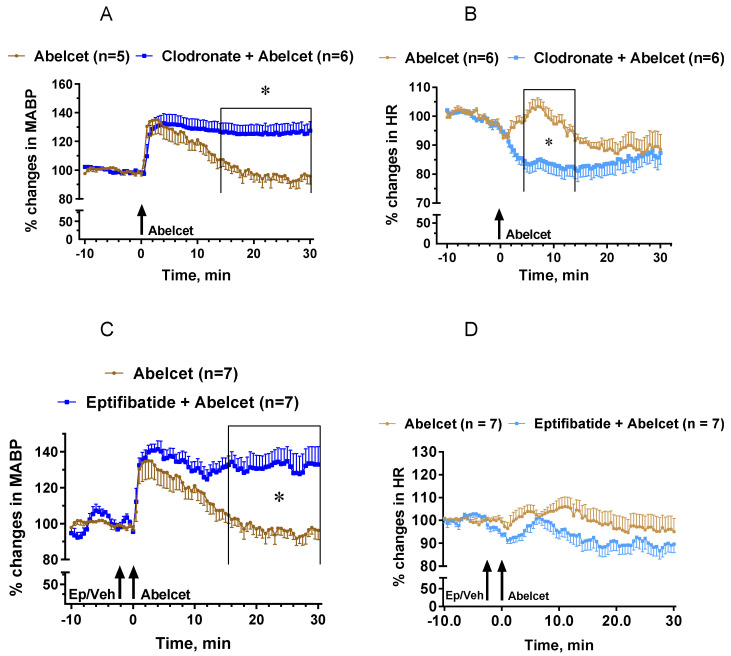
Effects of macrophage depletion with clodronate liposomes and platelet glycoprotein IIb/IIIa receptor inhibition with eptifibatide on the Abelcet-induced changes in MABP and HR in anesthetized NMRI mice. The time of treatment with eptifibatide or its vehicle and Abelcet injection is indicated by arrows. Clodronate or empty liposomes were injected via the tail vein at 200 mL/mouse (containing 1 mg clodronate) two days before the experiment. (**A**,**C**) Mean arterial blood pressure (MABP); (**B**,**D**) heart rate (HR). (**A**,**B**) Macrophage depletion with clodronate liposomes; (**C**,**D**) treatment with eptifibatide. Veh: vehicle. * *p* < 0.05. Vehicle- and clodronate- and eptifibatide-treated groups were compared using two-way ANOVA for repeated measurements followed by Dunnet’s multiple comparison tests. Macrophage depletion with clodronate liposomes lengthened the increase in MABP from min 12 after Abelcet administration, and decreased HR from 4 to 14 min after Abelcet administration. Treatment with eptifibatide lengthened the increase in MABP from 16 min after Abelcet administration but did not alter HR.

Mimicking the effect of the C5aR blocker, macrophage depletion with clodronate liposomes lengthened the MABP response to Abelcet, as MABP remained elevated up to 30 min after Abelcet administration (Figure 4A). Moreover, HR decreased from 4 to 14 min after treatment with Abelcet in the macrophage-depleted group compared to the control group (Figure 4B). Macrophage depletion had an effect similar to C3a, causing bradyphylaxis in MABP; however, unlike the receptor inhibitor, it also had an impact on HR, causing bradycardia. Inhibition of platelet activation with eptifibatide, a platelet glycoprotein IIb/IIIa receptor inhibitor, also extended the Abelcet-induced hypertension as MABP remained elevated up to 30 min after treatment with Abelcet (Figure 4C). However, HR was similar in the two groups (Figure 4D).

### 3.5. Effects of Abelcet in COX-1- and TP-Deficient Mice with and without Complement Depletion

**Figure 5:**
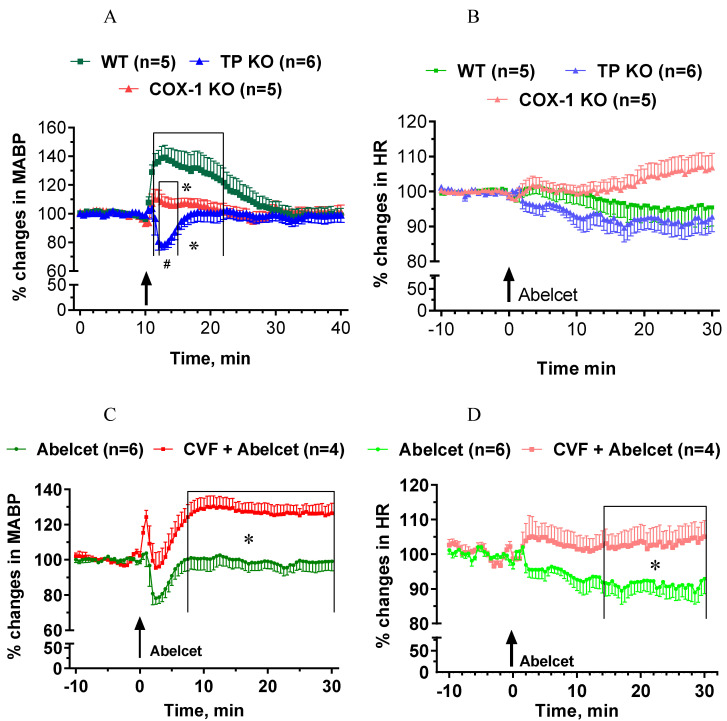
Effects of Abelcet on MABP and HR in anesthetized, control a complement depleted cyclooxygenase-1 (COX-1) or thromboxane prostanoid receptor (TP)-deficient and wild type (WT) C57BL/6N mice. (**A**,**C**) Effects of Abelcet on mean arterial blood pressure (MABP) and (**B**,**D**) on heart rate (HR) in COX-1- or TP-deficient and WT C57BL/6N mice. (**A**,**B**) Effects of Abelcet on MABP and HR in complement depleted TP-deficient and WT C57BL/6N mice. Complement depletion was accomplished with pretreatment with cobra venom factor (CVF). * *p* < 0.05 vs. wild type (WT) mice; # *p* < 0.05 between TP- and COX-1-deficient mice. WT and TP- or COX-1-deficient or control and complement depleted groups were compared using two-way ANOVA for repeated measurements followed by Dunnet’s multiple comparison tests. The effects of Abelcet on mean arterial blood pressure (MABP) was almost fully abolished in COX-1-deficient mice but heart rate (HR) was not affected. The Abelcet-induced hypertension was reverted to a transient hypotension in TP-deficient mice but heart rate (HR) was not affected. Depletion of complement with CVF caused a delayed hypertension in TP-deficient mice from 8 min, and HR was also significantly increased from 14 min after Abelcet administration.

The Abelcet-induced hypertension was fully abolished in COX-1-deficient mice (Figure 5A), while Abelcet caused a very short-lived hypotension in TP-deficient mice compared to COX-1-deficient mice (Figure 5A). HR was similar in the three groups, although a small tendency for an increase in HR was observed in the COX-1-deficient mice (Figure 5B). Upon complement depletion Abelcet caused a delayed hypertension in TP-deficient mice (Figure 5C); an effect similar to that seen in WT mice and increased HR from 14 min after Abelcet administration (Figure 5D).

## 4. Discussion

### 4.1. The Role of Complement Activation in Anaphylactoid Reaction in Mice

As described previously, intravenous administration of Abelcet, an amphotericin B-containing liposomal preparation, caused an anaphylactoid reaction in mice characterized by thrombocytopenia, leukocytosis and increased plasma C3a and thromboxane concentration and caused a transient hypertension [2]. The current study revealed that the Abelcet-induced hypertension has two phases. The early phase is mediated by COX-1-derived prostanoids, likely TXA2. The late phase is not TP/TXA2-dependent. 

It seems clear that the early phase of the hypertensive effect is mediated by cyclooxygenase products as it was fully abolished in COX-1-deficient mice. The main effector of the early hypertensive effect is likely TXA2, as no increase in MABP was observed in TP-deficient mice; on the contrary, MABP decreased for a few min after Abelcet administration. This short-lived hypotensive effect seems to be caused by vasodilatory cyclooxygenase products, such as prostaglandin E2 [18], PGD2 [19], and/or prostacyclin (PGI2) [20]. The prominent role of TXA2 is supported by our previous findings that both amphotericin B containing liposomes and direct C activators increased plasma TXB2 concentration in both mice and rats [2,12,20,21]. Furthermore, in a preliminary study we have also shown that the same C3a peptide, also used in the current experiments, induced an endothelium-independent vasoconstriction in the mouse aorta, in vitro, that was abolished in TP-deficient animals, i.e., it was also mediated by vasoconstrictor prostanoids, likely TXA2 [17]. The source of TXA2 can mainly be the vasculature as inhibition of platelets and macrophages did not alter the first phase of the hypertensive effect of Abelcet. It seems also obvious that the C system has only a small contribution to the release of TXA2 as the TP-dependent early phase of the hypertensive effect was hardly altered by C depletion, and it was attenuated by C3aR blockade to a small extent only. Therefore, the early phase of the Abelcet-induced hypertensive effect is mainly CIPA, i.e., it is more or less a C-independent reaction [2].

Quite surprisingly, complement depletion with CVF and C5aR inhibition uncovered a yet unknown late phase of hypertension upon Abelcet administration. We have no explanation for the mechanism of the late phase, as it remains hidden by C activation [2]. However, the current experiments revealed that C activation has a major role in the reversal of the late phase as both complement depletion and C5aR inhibition transformed the Abelcet-induced transient hypertension to a long-lasting one. The delayed hypertension was independent of TXA2 as it was clearly seen in complement depleted TP-deficient mice. It is important to note that C depletion with CVF did not alter the pharmacokinetics of liposomes, i.e., longer hypertension cannot be the consequence of longer circulation time of liposomes [21]. The late hypertensive effect seems to be supported by the finding that the second administration of Abelcet caused an increase in MABP, i.e., the second liberation of TXA2 exaggerated the late hypertensive response to some extent.

### 4.2. The Roles of Platelets and Macrophages 

Our current results indicate that vasoactive mediators released from macrophages and platelets restored blood pressure to normal during the late phase of hypertension. These results are compatible with previous findings that macrophages and platelets are important cellular mediators of anaphylaxis in mice [22,23,24]. These results seem to suggest that the main feature of the mouse anaphylaxis is a long-lasting hypertension that is terminated by vasoactive mediator released from macrophages and platelets, which were activated with some delay. In other words, inhibition of platelets and macrophages exposed the late phase of hypertension similarly to C depletion and C5aR blockade, suggesting that these cell types released vasodilator mediators. Since both C depletion/C5aR blockade and platelet/macrophage inhibition had similar effects, it is reasonable to suggest that the release of vasodilator mediators was mediated at least in part by Ca5R. 

Platelets and macrophages can mutually regulate each other’s functions at least during inflammation [25,26,27,28,29,30]. The great number of mediators released by these effector cells forms a complex chain of events. Blockade of a certain player can interfere with this system that can be an explanation why inhibition of platelets and macrophages has the same effect. For this reason, it is rather difficult to identify the main effector vasoactive mediators either experimentally or theoretically. This task is even more difficult as the anaphylactoid reaction and their mediators increase blood pressure in the mouse, while hypotension is observed in many other species like in rats [5,20,21]. However, LPS-induced anaphylaxis causes hypotension in mice as well [31]. However, if the dose is high, as in the case of zymosan and CVF in our previous study [2], a transient hypertension can be followed by hypotensive shock, i.e., high doses of any medication capable of causing infusion reaction may induce anaphylaxis at high doses.

The main mediators of allergy are histamine [32], platelet-activating factor (PAF) [33], but prostaglandins [34], leukotrienes [35], and serotonin [32] can also contribute to mediate the vascular effects of pseudoallergy. PAF is released by both platelets and macrophages [36,37], so PAF can be one of the vasoactive mediators that decreases blood pressure during the late phase of hypertension. 

Previous studies also support a role for macrophages and platelets in passive systemic anaphylaxis. Monocyte/macrophage depletion with clodronate liposomes also abolished the drop in body temperature, as a measure of the IgG-induced anaphylaxis mediated via the classical complement pathway [23].

### 4.3. Limitations

We tested that CVF fully depleted plasma complement activity. However, the effect of clodronate liposomes on the types of white blood cell and the extent of changes were not tested. However, we showed that no Kupffer cells were detected histologically in the liver after treatment with clodronate liposomes [17]. Moreover, blockade of glycoprotein IIb/IIIa receptors using eptifibatide may not fully abolish platelet activity [38], so platelet activation via other receptors could have a small contribution to the HSR observed during the study even after eptifibatide administration. Further studies are needed to confirm the translational relevance of our observations because blood pressure increases in mice [2], but blood pressure decreases in rats [12,20,21], and blood pressure changes are less frequent in pigs [3] during pseudoallergy.

## 5. Conclusions

The current results indicate that the most characteristic feature of the Abelcet-induced anaphylactoid reaction is a large increase in systemic arterial pressure. The hypertensive effect has two phases, an early phase, which is mainly mediated by TXA2 with a small contribution of the complement system, and a second phase, which is reversed by complement activation mainly via the C5aR. The vasodilatory mediators are released from macrophages and platelets during the second phase. These results underline our previous observations that the anaphylactoid reaction is a complex mechanism which has complement-dependent and also complement-independent mediators.

## Data Availability

All data generated during this study can be obtained from Gábor Szénási (szenasi.gabor@med.semmelweis-univ.hu) upon request.
